# *Mycobacterium bovis* Infection in Animals and Humans, 2nd Edition

**DOI:** 10.3201/eid1208.060526

**Published:** 2006-08

**Authors:** Mitchell V. Palmer

**Affiliations:** * US Department of Agriculture, Ames, Iowa, USA

**Keywords:** *Mycobacterium bovis* Infection, book review, Mitchell Palmer, M. bovis, M. bovis epidemiology

The stated purpose of the second edition of Mycobacterium bovis Infection in Animals and Humans is to provide medical professionals, allied health scientists, research workers, and graduate students with current information on the significance of M. bovis in the control and eradication of tuberculosis in animals and humans. This newest edition deals with topics such as the public health significance of M. bovis, pathogenesis of M. bovis, epidemiology of M. bovis (with an entire chapter on molecular epidemiologic techniques), PCR detection of M. bovis with formalin-fixed tissues, and DNA vaccines. As with the first edition, the second edition delivers several updates from various countries on the status of M. bovis infection in animals and humans. Little accessible published information has been available on this topic, which makes the book especially useful.

The second edition also deals with several areas not covered in the first edition, including molecular epidemiology, evolution of the M. tuberculosis complex, tuberculosis caused by M. pinnipedii in fur seals and sea lions, the economics of bovine tuberculosis, and cost-benefit analysis of disease eradication programs. Several chapters deal with timely issues related to tuberculosis in wildlife.

In spite of its strengths, the second edition adds little additional information to material provided in the first edition on the topics of pathogenesis or diagnosis of bovine tuberculosis. In addition, although most of the photomicrographs are adequate, several are of such poor quality that they are of little use. Also, as with any multiauthored volume, some repetition occurs on general topics. The book achieves its stated purposes, however, and will be especially useful as a reference for researchers, regulatory agencies, and graduate students. It will be less informative for those interested in detailed discussions on research in the field of pathogenesis or diagnosis of M. bovis infection.

